# The Impact of Self-Consistency Congruence on Non-Suicidal Self-Injury in College Students: The Mediating Role of Negative Emotion and the Moderating Role of Gender

**DOI:** 10.3390/ijerph191911898

**Published:** 2022-09-20

**Authors:** Yang Li, Keke He, Changfeng Xue, Chun Li, Chuanhua Gu

**Affiliations:** 1 Mental Health Education Center and Education Development Research Institute, Nanchang University, Nanchang 330038, China; 2 School of Psychology, Central China Normal University, Wuhan 430000, China; 3 Department of Preschool Education, Qingdao University, Qingdao 266000, China

**Keywords:** self-consistency congruence, negative emotion, gender, non-suicidal self-injury

## Abstract

Non-suicidal self-injury (NSSI) can be defined as the deliberate destruction of body tissues to generate harm. College students have a higher incidence of NSSI. With the deepening of research on college students’ NSSI, the connection between their self-consistency congruence and NSSI has drawn the attention of many scholars. The current study examined the association between self-concordance and NSSI, the mediating function of negative emotions, and the moderating role of gender. We surveyed 1020 college students from three universities in Jiangxi Province using a self-concordant scale, a NSSI questionnaire, and a negative emotion questionnaire. The results showed that self-concordance was negatively correlated with NSSI. There is an obvious negative connection between self-consistency congruence and negative emotions. There was a significant positive correlation between negative emotions and the NSSI scores. Negative emotions could mediate the association between self-consistency congruence and NSSI. Compared to males, females’ self-concordant effects on negative emotions are easier to moderate.

## 1. Introduction

In 2017, the World Health Organization stated that suicide was the second major cause of death among young people aged 15–29 years worldwide [1], and non-suicidal self-injury (NSSI) was the strongest predictor of suicidal behavior [2,3]. NSSI is related to serious negative consequences and has become an increasingly severe global public health issue [4].

Recently, several researchers have focused on NSSI and its causes [5], characteristics [6], effects on mental health [7], differences in age and gender [8], assessment and treatment [9], relationship with personality traits [10], and relationship with childhood trauma have been investigated [11].

According to previous studies on NSSI, this behavior mostly begins in adolescence [12]. In addition, the second peak of NSSI occurs in young adulthood, that is, around the age of 20 to 24, which, for many people, accords with college years [13]. For many people, going to college is a turning point in the transition from adolescence to adulthood [14]. Although this period tends to be surprising and exciting, there is often significant instability in terms of employment, housing, finances, and relationships [15]. As a result, it is an especially vulnerable and sensitive time with a high incidence of mental health problems, including suicide and self-injury [16,17]. According to an assessment report of NSSI in a sample of Belgian college students, the occurrence of NSSI among college students within one year was 10.3% [18].

Different studies have reported different views regarding the pathological mechanisms of NSSI. The results of an existing meta-analysis showed that the experience of being bullied was highly associated with the occurrence of NSSI, and adolescents who were bullied were 2.41 times more likely to experience NSSI than those who were not bullied [19]. Several studies have shown that self-criticism is significantly correlated with NSSI frequency [20,21]. Self-injurers reported obviously higher levels of self-criticism than non-injurers [22], and self-criticism significantly predicted NSSI in adolescents [23]. An empirical avoidance model of NSSI shows that low self-esteem is an unfavorable state for individuals, who may hope to alleviate this state through NSSI [24]. Through an analysis of previous studies, the moderating effect between NSSI and self-consistency congruence (under which conditions the influence of NSSI on self-consistency congruence becomes stronger or weaker) deserves further analysis. Therefore, the current study explored the impact of college students’ NSSI on self-consistency congruence as well as its mechanisms.

A study on the association between self-consistency congruence and anxiety traits found a negative connection between self-consistency congruence and anxiety [25]. To some extent, this finding demonstrates a strong correlation between self-consistency congruence and NSSI. However, the mediation mechanisms that explain this correlation remain unknown. To fill the existing gap, this study attempted to build a moderated mediation model to investigate the mediating function of negative emotion between self-consistency congruence and NSSI as well as the moderating function of gender between the two.

### 1.1. Non-Suicidal Self-Injury (NSSI)

NSSI can be defined as the deliberate destruction of one’s own body tissue, causing injury [26]. Methods include hitting, cutting, burning, smashing, and scratching one’s body [27]. This behavior has also been found to be socially disapproved [28,29]. Researchers worldwide have mainly discussed the causes of adolescent NSSI based on interpersonal and personal factors. Interpersonal factors include early abuse or neglect [30], attachment [31], and social support. Personal factors include emotional regulation, self-efficacy, and self-esteem [32]. Low levels of parent–child attachment make individuals more likely to produce or even reinforce negative emotional experiences such as guilt, frustration, shame, and self-rejection, which further increases the risk of negative behaviors, such as NSSI [33]. Parent–child attachment significantly negatively predicts NSSI. Childhood maltreatment is also a predictor of NSSI [34], and domestic data demonstrate that the risk of NSSI in middle school students with childhood abuse experience is 1.63 times higher than that in students without childhood abuse experience [25]. Li et al. (2021) found an obvious positive association between child sexual abuse and NSSI [35], suggesting that students who have been sexually abused in childhood are at a greater risk of NSSI thoughts and behaviors. Forreste et al. (2017) believed that adolescents’ NSSI was related to self-esteem, and the lower the self-esteem level, the more likely the occurrence of NSSI [33]. Qin et al. (2017) also found that NSSI can regulate emotions, relieve emotions such as high shame tendency, high anxiety, and high guilt, and release angry and impulsive emotions caused by bullying [36].

Currently, the frequency of NSSI among college students is increasing annually, especially in China, the country with the largest number of college students in the world. Pan et al. (2016) performed a meta-analysis on the detection rate of NSSI among Chinese college students by screening 23 studies on the subject; the combined detection rate of NSSI was 16.6% [37]. The detection rate was higher in comparison with that stated in the United Kingdom (6.9%) and Germany (14.9%). Many studies have noted the importance of NSSI in mental health problems among college students, mainly by investigating the role of family and school, such as parental psychological control [38] and negative attitudes toward school [39]. There are also studies on the individual factors that influence NSSI. Domestic and foreign studies show that personality and emotion have a clear impact on NSSI, and the influencing factors include self-consistency congruence and negative emotion [40,41].

### 1.2. Self-Consistency Congruence

Self-congruence is one of the most vital concepts in Rogers’ therapy, personality theory, and interpersonal correlation theory. Self-consistency congruence denotes the internal consistency of an individual, as well as the coordination between the self and experience, containing self-evaluation and self-consistency congruence of ability and emotion. Personal self-consistency congruence is closely related to mental health. Self-disharmony makes people feel anxious and nervous. In addition, if an individual experiences a gap between themselves and their own experience, this gap will lead to a state of disharmony [42]. The disharmony between the self and experience can be a vital factor in generating psychological disorders [37].

There is a close association between self-consistency congruence and mental health [43]. Studies have shown that college students’ self-harmony-self-consistency congruence is negatively correlated with NSSI, indicating that the lower the degree of self-consistency congruence, the higher the incidence of NSSI [40]. Simultaneously, self-consistency congruency affects individual emotions, and differences in self-consistency congruence can lead to changes in emotions [44].

### 1.3. The Mediating Role of Negative Emotion

Ekman (1992) proposed the classical theory of basic emotions, describing them as widely shared natural and instinctive psychological states including happiness, anger, and sadness. Essentially [45], emotion is an indirect feedback system invoking the process of cognitive reflection associated with individual values and guiding future behaviors [46]. Feelings of pain and anxiety or negative self-directed judgments and ruminations are negative emotions experienced by many people. When these feelings are more frequent or intense and interfere with everyday functioning, people face emotional disorders including anxiety and depression [1]. To investigate the mediating role of negative emotions between NSSI and self-consistency congruence, this study drew on the negative emotions determined by Harmon-Jones et al. (2016) in terms of neurological, biological, expressive, and interactive characteristics, including anger, disgust, fear, anxiety, and sadness [47]. Studies have shown that these emotions can seriously affect not only the mental health of college students but also their physical health [48].

Rogers (1959) believed that if one’s actual experience is close to one’s perceived ideal state, then one is closer to the state of self-consistency congruence. A contrast between experience and the ideal state is perceived as dissonant, which causes individuals to experience negative emotions, dissonance, and psychological distress, and this affects the entire range of future behavior. The empirical avoidance model shows that NSSI individuals diverts attention to NSSI behaviors and helps individuals manage or regulate their emotions [24]. Emotional cascade models also suggest that experiencing high levels of negative emotions can increase the likelihood of adopting NSSI as a distraction [49,50]. Adolescents may use NSSI as a coping strategy to regulate their antipathy experiences [51]. Salters-Pedneault, Tull, and Roemer (2004) also proposed that when individuals cannot adapt to negative emotions and choose to control or avoid them, negative emotions may progressively accumulate and finally rebound, resulting in even stronger negative emotions [52]. Ultimately, the possibility of NSSI increases [53]. Studies have shown that college students’ self-consistency congruence is negatively correlated with their NSSI and negative emotions. Therefore, we hypothesized that negative emotions may be a vital mediator between self-consistency congruence and NSSI. To date, no study has verified whether negative emotions mediate the association between self-consistency congruence and NSSI among college students. However, the literature provides preliminary support for this mediation process.

First, self-dissonance may reinforce negative emotions. Some early studies confirmed that inconsistencies between the internal self and external experience are associated with diverse forms of psychological distress [54]. Emotional problems also affect the social and psychological development of adolescents, thus negatively affecting the development of their self-identity [55]. Different negative thoughts (such as “I feel disappointed in myself”, “I feel guilty”) add meaning to experiencing negative emotions [56]. Negative emotions are caused by the perceived gap between the actual self and ideal self, or the perceived gap between the actual self and ought self [57]. In an earlier study, Dent and Teasdale found that self-deprecation leads to negative emotions [58]. Clearly, there is a very close relationship between self-consistency congruence and negative emotions.

Negative emotions may lead to NSSI, which in turn regulates the negative emotions associated with NSSI. Existing ecological momentary assessment research results support the view that people often use NSSI to regulate emotions, particularly to reduce negative emotions [59,60]. For example, people who engage in NSSI often report an unpleasant emotional state before NSSI, as well as enhanced emotions and lowered negative emotions after participating in these behaviors [61]. This generally indicates that the rise of negative emotions usually occurs before the onset of NSSI impulse and behavior, and that negative emotions usually decrease after NSSI [62,63]. In addition, high levels of self-reported negative urgency predict the onset of NSSI among college students [64], and compared to individuals with low negative emotions, individuals with higher negative emotions are more prone to NSSI and repeated NSSI [65].

Based on the above literature review, we posit that negative emotions play a mediating role in the association between self-concordance and NSSI.

### 1.4. The Moderating Role of Gender

Many studies have been conducted on gender differences in NSSI; however, inconsistent conclusions have been drawn. Barrocas (2012) and Sornberger et al. (2012) indicated that NSSI is generally considered more common in women [66]. Other studies have demonstrated that the prevalence of NSSI may be the same in men and women [67]. Based on this research condition, this study explored gender differences in college students’ NSSI and further investigated whether gender differences exert a regulatory function in the effects of self-consistency congruence and negative emotions on college students’ NSSI. Although no studies have tested this hypothesis, there is preliminary support for it in the literature. Additionally, studies have shown that with the improvement of the level of self-consistency congruence, female college students’ negative emotions enhance more slowly when compared with those of male college students [68]. Another study noted that men are happier than women, and with an increase in negative emotions, women are more likely than men to engage in NSSI [69].

### 1.5. The Present Study

**Figure 1:**
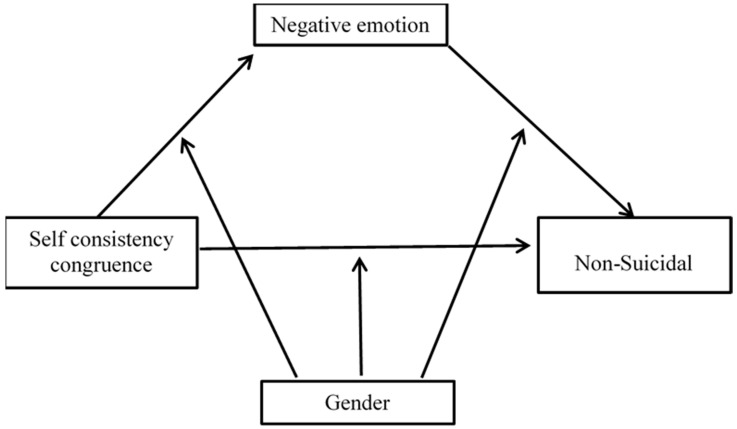
Moderated mediation model.

This study investigated whether negative emotions facilitate the correlation between self-concordance and college students’ NSSI and whether gender moderates the direct and/or indirect association between self-concordance and college students’ NSSI (Figure 1).

## 2. Methods

### 2.1. Participants and Procedure

By adopting the overall sampling approach, researchers chose three universities from Jiangxi Province for the questionnaire survey. In total, the distribution of 1020 questionnaires was performed; 1007 questionnaires were gathered with 993 valid questionnaires being returned, among which 616 (62%, *M*_age_ = 18.60, *SD* = 0.60) were finished by boys and 377 (50.6%, *M*_age_ = 18.50, *SD* = 0.50) by girls; Among them, 282 (28.4%, *M*_age_ = 18.70, *SD* = 0.60) are only children and 711 (71.6%, *M*_age_ = 18.60, *SD* = 0.50) are not only children.

The approval of the current work was acquired from the Jiangxi Educational Science Planning Project. Prior to the data collection, written informed consent forms from participants were obtained, including a simple description for the research. Participants were guaranteed of the confidentiality of information and name, and the answers would be used only for research. Participants were allowed to quit the research at any time without penalty. Next, the participants completed questionnaires distributed in the same order. Well-trained postgraduate students were responsible for implementing the measures and collecting brief biographical data. It took around 30 min to fill in the questionnaire.

### 2.2. Measures

#### 2.2.1. Self-Consistency Congruence Scale

The self-consistency congruence scale of Wang (1994) referred to Rogers’ seven dimensions [70], which was designed based on the assessment of the therapist and the subject [71]. The scale consisted of “Dissonance between self and experience” (16 items), “Flexibility of self” (12 items) as well as “Stereotypicality of self” (7 items) dimensions. Moreover, items were rated on five levels from “not at all” to “completely”. In addition, the scale used reverse scoring, in which scores of items in all dimensions were summed to obtain factor scores, the self-flexibility score was reversed, and the sum of the three factors indicated the total score of self-consistency congruence. In addition, the total score was in the interval of 35–175, and the higher the score, the lower the Self-Consistency Congruence level. Following the expression habits, in the data analysis, “self-disharmony between self and experience” and “self-rigidity” scores were reversed, whereas “self-flexibility” score was not reversed, and the scores of all dimensions were summed to obtain the total score. Higher scores suggested higher self-harmony levels. The internal consistency coefficient for the scale reached 0.85, with factors of 0.50–0.76. Apart from that, the Pearson correlation with the factors and the total scale was 0.40–0.49 and 0.62–0.73, separately. Moreover, the scale shows favorable reliability as well as validity in multiple reports.

#### 2.2.2. Non-Suicidal Self-Injury Questionnaire

This questionnaire was revised by Feng Yu on the basis of Zheng Ying’s NSSI questionnaire based on Grace’s definition of NSSI. The questionnaire refers to the life event scale, which divides the impact of an event into two dimensions, frequency and degree, and consists of 18 items and an open item (only 9 students filled in this item in this study, so only the first 18 items were analyzed in data analysis). The evaluation of NSSI for each item is divided into 4 levels: level 0 (0 times), level 1 (1 time), level 2 (2–4 times) and level 3 (5 times or more). If the answer is 0–3, the score is 0–3. The degree of injury can be divided into five grades according to the individual’s subjective feelings: none, mild, moderate, severe and very severe. “None” is 0 points, “mild” is 1 point, “moderate” is 2 points, “severe” is 3 points, and “very severe” is 4 points. NSSI score of each item is the product of frequency score and degree score. The sum of the scores of 18 items is used as the total score of NSSI. It indicates the higher the score is, the higher the level of NSSI is. In the current work, the internal consistency coefficient α of the questionnaire reached 0.88.

#### 2.2.3. Negative Emotion Questionnaire

Using Watson D (1988) and others develop positive negative emotions scale (The Positive Affect and Negative Affect Scale, PANAS). Furthermore, the original scale was composed of 20 adjectives used to describe positive or negative emotions. In this scale, only 10 questions (questions 2, 4, 6, 7, 8, 11, 13, 15, 18 and 20) of negative emotions were selected to test the subjects. The subjects were asked to use the likert 5-point scoring method to score from 1 (almost none) to 5 (extremely much) according to their actual situation in the past 2 weeks. According to Huang Li et al. (2003) the Cronbach’s α coefficient of 10 items of negative emotion was 0.83 when PANAS was tested in Chinese population in Chinese version.

### 2.3. Data Analysis

We adopted SPSS 22.0 as well as SPSS macro-PROCESS for analyzing the data (Hayes, 2013). At first, descriptive statistics were initially evaluated, followed by computing Pearson’s correlation coefficients with the purpose of assessing the correlations among the variables. In the end, PROCESS (Model 4 and Model 59) was employed to examine the multiple-mediation models involving self-consistency congruence and NSSI based on the correlation of negative emotion and gender.

## 3. Results

### 3.1. Common Method Deviation Test

We adopted Harman one-factor test for testing common method bias. Additionally, the findings revealed 5 factors with characteristic roots greater than 1, accounting for 58.28% of the total variance. In addition, the amount of variance accounted for by the first factor was 33.02%, on the basis of L. R. Long’s criterion, with the critical criterion being <40%, implying that there existed no serious common method bias variance.

### 3.2. Primary Analysis

Table 1 presents the mean, standard deviation as well as correlation coefficient matrices for all of the variables. Self-consistency congruence scores were significantly negatively correlated with negative emotion; negative emotion was positively correlated with NSSI; and self-consistency congruence scores showed negative relationship to NSSI.

### 3.3. Intermediary Model Test with Moderation

Initially, with the purpose of testing the mediating function of negative emotion between self-consistency congruence and college student NSSI, a mediation impact analysis was carried out with Model 4 in the PROCESS program proposed by Hayes. According to the obtained findings, self-consistency congruence scores could negatively predict adolescent NSSI (*β* = −0.0961, *t* = −2.8576, *p* < 0.01) and negatively predict negative emotion (*β* = −0.2140, *t* = −14.7934, *p* < 0.01), and negative emotion positively predicted NSSI (*β* = 0.3681, *t* = 5.5707, *p* < 0.01). Bootstrap 95% confidence intervals for both the direct impact of self-consistency congruence on NSSI and the mediating effect of negative emotion did not include 0 (see Table 2), implying that negative emotion, to some extent, can regulate the impact between self-consistency congruence and NSSI. In addition, the direct impact (−0.0961) and mediating impact (−0.0788) occupied 53.5% and 46.5% of the total effect (−0.1749), respectively. 

The mediated model with moderation was explored by adopting Model 59. The results can be seen in Table 3. After gender was included in the model, the self-consistency congruence score clearly and negatively predicted negative emotion (*β* = −0.2187, *t* = −14.9805, *p* < 0.01). Gender was the notable predictor of negative emotion (*β* = 0.8739, *t* = 2.1968, *p* < 0.05). Furthermore, the interaction item for the self-consistency congruence score and gender was not regarded as a significant negative predictor of well-being (*β* = −0.0244, *t* = −0.7953, *p* > 0.05). The self-consistency congruence score referred to a significantly negative predictor of NSSI (*β* = −0.094, *t* = −2.6301, *p* < 0.01). Gender was not a negative predictor of NSSI (*β* = −0.4720, *t* = −0.5579, *p* > 0.05) while negative emotion was a significant predictor of NSSI (*β* = −0.3629, *t* = −9.0732, *p* < 0.01). Notwithstanding, the interaction item of the self-consistency congruence score and gender was not the significant predictor of NSSI (*β* = 0.0303, *t* = 0.4181, *p* > 0.05). The interaction item of negative emotion and gender was the significant predictor of NSSI (*β* = −0.3975, *t* = −2.7740, *p* < 0.01).

**Figure 2:**
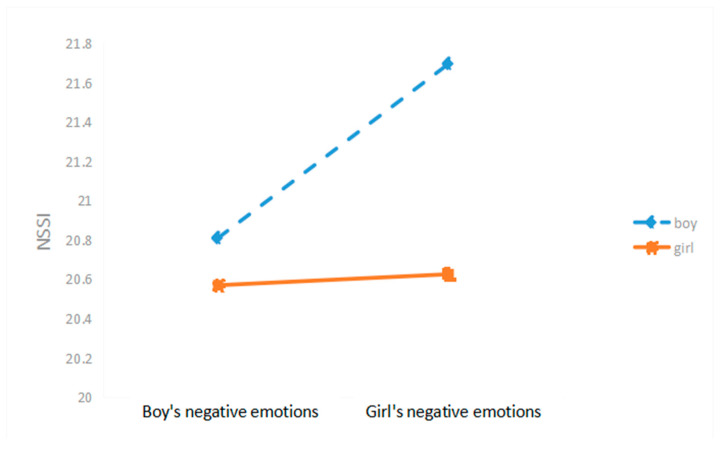
Moderating Role of Gender.

Moreover, we carried out a simple slope analysis. The positively predictive effect of adolescent boys’ negative emotion scores on NSSI was of significance (*β* = −0.3797, *t* = 7.7139, *p* < 0.01) with the predictive impact of adolescent girls’ negative emotion scores on NSSI being not significant (*β* = 0.6203, *t* = 1.5874, *p* > 0.05); in comparison to girls, as negative emotion scores enhanced, boy’s NSSI increased more rapidly. The results are presented in Figure 2.

## 4. Discussion

### 4.1. The Impact of Self-Consistency Congruence on College Students’ Non-Suicidal Self-Injury

The results demonstrated that self-concordance could significantly negatively predict college students’ NSSI; the higher the self-concordance, the lower the probability of NSSI.

Self-identity is determined by an individual’s recognition of their qualities and characteristics, especially with regard to the social environment [72]. An existing study found that adolescents’ self-concordance generates a significant negative predictive impact on NSSI, and the higher the self-concordance level, the less likely NSSI is [35]. An individual’s attitude and experience toward their body also play a core role in the development of self-identity [73,74], and negative attitudes toward one’s body may lead to difficulties in self-identity [75,76]. These results indicate that self-loss can generate NSSI. Individuals with low self-concordance will have higher self-denial and will tend to exhibit NSSI behavior. Therefore, low self-concordance levels are an important inducing factor for college students’ NSSI.

### 4.2. Analysis of the Mediating Impact of Negative Emotion

The results showed that self-concordance negatively predicted negative emotions, negative emotions positively predicted NSSI, and negative emotions played a mediating role between self-concordance and NSSI.

Emotion is defined as a psychological state in which individuals evaluate stimuli meaningful to their goals and experience negative or positive emotions. Important individual differences exist in the degree of easy activation, intensity, and persistence of individuals’ emotional tendencies [77]. Emotional problems showed an important association with self-identity. Most adolescents with emotional problems—normal, marginal, or abnormal—experience delayed identity status. Mood dysplasia can cause behavioral issues and influence self-identity development, and existing studies show that adolescents who experience borderline and abnormal emotional problems often feel sad, depressed, disappointed, anxious, stressed, or depressed [55].

Individuals with a high level of self-harmony often experience ease and pleasure because of the harmony between the internal consistency of the individual self and the external experience of the self. Nevertheless, individuals with low levels of self-harmony tend to experience negative emotions because some of their self- and external experiences conflict, generating negative emotions.

In addition, there is a close relationship between negative emotions and NSSI. Studies have shown that compared with individuals without NSSI, individuals with NSSI show higher levels of negative emotions in self-recall reports and diaries [78]. According to the general stress theory proposed by Agnew (1992), stimulating events or situations that make people nervous may trigger negative emotions including anxiety, anger, and depression [79]. To cope with these negative emotions, individuals may either attack or injure themselves. Existing research also shows that negative emotions can positively predict the occurrence of NSSI [80]. Although individuals with negative emotions relieve and release their emotions through NSSI, they also increase their tolerance to pain and the pain threshold through white injury [81].

This study found that negative emotions had positive predictive effects on college students’ NSSI scores. When individuals’ negative emotions increase, they experience a higher frequency of NSSI, which is consistent with previous research findings [82].

### 4.3. Analysis of the Moderating Effect of Gender

Gender has been shown to exert a moderating effect on the association between negative emotions and NSSI.

As women are more likely to express emotions than men [83] and men are more likely to experience difficulty in emotional regulation than women [84,85], girls experience more intense negative emotions than boys when their self-harmony level is reduced during adolescence. Međedovi’c et al. also found that the emotional and interpersonal aspects of mental illness predicted greater emotional distress in women than in men [86]. Whalen et al. demonstrated that the prevalence of depression in girls is higher than that in boys [87]. In the study samples, Kingsbury et al. found that there were significantly more females who experience thoughts of NSSI (12.4%) and females with NSSI (5.3%) than males (5.3% and 2.3%, respectively) [88]. Adolescent girls experienced a faster decline in positive emotions when their self-concordance level was lower than that of boys. When girls faced internal and external self-conflict and low self-concordance, they had a stronger negative emotional experience than boys. This indicates that girls with low self-consistency congruence levels should obtain more focus, support, and protection during adolescent mental health education and maintenance [35]. Therefore, as women’s positive emotions decline, their negative emotions enhance faster than men’s, and they have a greater frequency of NSSI actions.

In this study, the relationship between negative emotions and self-injury has been discussed to address researchers’ recent increase in focus on NSSI and on the relationship between self-consistency congruence and self-injury. However, the role of gender as a demographic variable in self-injury, self-consistency congruence, and negative emotions had not been explored to date. This study is the first to explore the moderating role of gender in the relationship between self-injury and negative emotions, filling a gap in this field. More importantly, our findings also highlight the importance of considering gender equality and developing initiatives that target men’s mental health. Whether in daily life or in the process of education, men should improve their self-care and strive to find ways to relieve negative emotions to reduce their rate of self-injury.

## 5. Limitations and Future Research

Although we conducted in-depth research on the association between self-consistency congruence and NSSI among college students, there are still some limitations. First, although our study is beneficial in comprehending the causes of NSSI among college students, we cannot verify a causal relationship because of the cross-sectional design adopted in our study. Incorporating experimental or longitudinal designs in subsequent studies has greater potential to determine possible causal relationships between these variables. Second, although the participants were students from three institutions of higher learning in China, they may not completely represent all college students in this country. Therefore, the extent to which our results can be generalized to other populations remains unclear. Third, this study focused mainly on the internal factors of individuals, including self-consistency congruence and negative emotions. However, diverse external and environmental factors may also play a vital role. Future studies that examine both personal and environmental factors and use data from multiple sources may assist in further understanding the key determinants of NSSI among college students and how to handle it and minimize its negative effects. Fourth, the correlation between self-concordance and negative emotions, such as impulsive emotions, positive emotions, and body identity, should be examined in future studies. These factors were not included in the current study and should be explored in future studies. Finally, identity was mentioned many times in this research, but we did not study it in depth; however, we may conduct in-depth research on identity in the future.

## 6. Conclusions

To conclude, this study investigated a new perspective by examining other vital mediating factors between college students’ self-consistency congruence and NSSI. We found that negative emotions regulated the correlation between these two variables. Negative emotions exerted a partial mediating role, while gender moderated the relationship between NSSI and negative emotions. Negative emotion scores had a clear positive predictive impact on NSSI in adolescent boys. Compared to girls, boys’ NSSI increased faster with an increase in negative emotion scores. These results suggest that NSSI may be associated with self-consistency congruence and negative emotions and that gender differences exist in the association. Moreover, the current study offers ideas for self-concordant and negative emotional interventions for college students with NSSI.

## Figures and Tables

**Table 1:** Correlation analysis of variables.

Non-Suicidal Self-Injury	Mean	SD	Gender	Only Child	Self-Consistency Congruence	Negative Emotion	Non-Suicidal Self-Injury
Gender			1				
Only child			0.009	1			
Self-consistency congruence	120.5	13.2	0.066 *	−0.012	1		
Negative emotion	22.7	6.7	0.034	0.002	−0.420 **	1	
Non-suicidal self-injury	21.2	13.2	−0.018	−0.003	−0.174 **	0.227 **	1

* Indicates significant. ** Indicates extremely significant.

**Table 2:** Mediating role of negative emotions.

Non-Suicidal Self-Injury	Effect Value	Boot Standard Error	Boot CI Lower Limit	Boot CI Upper Limit	Relative Effect Value
Total effect	−0.1749	0.031	−0.2356	−0.1141	
Direct effect	−0.0961	0.0336	−0.0301	−0.0072	53.50%
The mediating effect of negative emotion	−0.0788	0.0377	−0.1681	−0.0302	46.50%

**Table 3:** Intermediary model with Moderating.

	Regression Equations		Overall Fit Index		Significance of Regression Coefficients	
Result Variables		R	R2	F	*β*	*t*
Negative emotion		0.4312	0.1860	75.3072 **		
	Self-consistency congruence (a)				−0.2187	−14.9805 **
	Gencer (b)				0.8739	2.1968
	A × b				0.0244	0.7953
Non-suicidal self-injury		0.2575	0.0663	14.0217 **		
	Self-consistency congruence (a)				0.094	−2.6301 **
	Gencer (b)				−0.4720	−0.5579
	negative emotion (c)				0.3629	−9.0732 **
	A × b				0.0303	0.4181
	C × b				0.3975	−2.7740 **

* Indicates significant. ** Indicates extremely significant.

## Data Availability

The data presented in this study are available on request from the corresponding author.
